# Accumulation of C-CTX1 in Muscle Tissue of Goldfish (*Carassius auratus*) by Dietary Experience

**DOI:** 10.3390/ani11010242

**Published:** 2021-01-19

**Authors:** Andres Sanchez-Henao, Natalia García-Álvarez, Daniel Padilla, María Ramos-Sosa, Freddy Silva Sergent, Antonio Fernández, Pablo Estévez, Ana Gago-Martínez, Jorge Diogène, Fernando Real

**Affiliations:** 1 Division of Fish Health and Pathology, University Institute of Animal Health and Food Safety (IUSA), University of Las Palmas de Gran Canaria, 35416 Arucas, Spain; julian.sanchez101@alu.ulpgc.es (A.S.-H.); daniel.padilla@ulpgc.es (D.P.); maria.ramos146@alu.ulpgc.es (M.R.-S.); freddy.silva101@alu.ulpgc.es (F.S.S.); antonio.fernandez@ulpgc.es (A.F.); fernando.real@ulpgc.es (F.R.); 2 Department of Analytical and Food Chemistry, Campus Universitario de Vigo, University of Vigo, 36310 Vigo, Spain; paestevez@uvigo.es (P.E.); anagago@uvigo.es (A.G.-M.); 3 Marine and Continental Waters Environmental Monitoring, IRTA, Ctra. Poble Nou, km 5.5, 43540 Sant Carles de la Ràpita, Spain; jorge.diogene@irta.cat

**Keywords:** ciguatera, Caribbean ciguatoxin, muscle bioaccumulation, cytotoxicity assay, goldfish, omnivorous fish, experimental model, detoxification

## Abstract

**Simple Summary:**

Some marine microalgae usually present in warm waters can produce ciguatoxins (CTXs); these toxins can accumulate in fish through the trophic chain, causing the food poisoning known as ciguatera in humans. It is important to understand how these compounds could be incorporated into fish muscle. For this purpose, this study was conducted using goldfish, an omnivorous freshwater species, daily fed raw fish flesh contaminated with a known toxicity concentration of CTX, seeking the accumulation profile in muscle and any signs of intoxication. Toxicity was detectable from day eight of the toxic diet and reached its maximum after two weeks. Signs of poisoning were observed after two weeks in all treated fish. However, two individuals developed strong symptoms, and one of them was separated and fed non-toxic food for 60 days; it showed recovery signs after the first week, and no toxicity was observed at the end of that non-toxic period. These results demonstrate that this toxin can accumulate in the muscle tissue of goldfish and produce associated symptomatology. Moreover, goldfish can recover and eliminate the CTX from its muscle if the toxin source is not available.

**Abstract:**

Ciguatoxins (CTXs) are produced by dinoflagellates usually present in tropical and subtropical waters. These toxins are bioaccumulated and transformed in fish causing ciguatera fish poisoning (CFP) in humans. Few trials have been performed to understand how CTXs are incorporated into fish. This study developed an experimental model of goldfish (*Carassius auratus*) fed flesh contaminated with Caribbean ciguatoxin (C-CTX1). Fourteen goldfish were fed 0.014 ng CTX1B (Eq. g^−1^ of body weight) daily, and control goldfish received non-toxic flesh. CTX presence was determined by a cell-based assay on days 1, 8, 15, 29, 36, 43, and 84. Toxicity was detected in muscle from the second sampling and then seemed to stabilize at ~0.03 ng CTX1B Eq. g^−1^. After two weeks, all experimental goldfish developed lethargy and loss of brightness, but only two of them displayed erratic swimming and jerking movements near the sixth sampling. One of these fish had its toxic diet replaced by commercial food for 60 more days; the fish showed recovery signs within the first weeks and no CTX activity was detected. These results indicate that C-CTX1 could accumulate in goldfish muscle tissue and produce toxic symptoms, but also remarked on the detoxification and recovery capacity of this species.

## 1. Introduction

Ciguatoxins (CTX) are a group of polyether compounds responsible for causing a worldwide illness known as ciguatera fish poisoning (CFP), which produces gastrointestinal, neurological, and cardiovascular symptoms [1]. CTXs have been traditionally divided into three groups according to the region where they were produced: P-CTX (Pacific Ocean), I-CTX (Indian Ocean), and C-CTX (Caribbean Sea) [2]. However, current nomenclature reflects the chemical diversity that divides them into three groups: oxopene (P-CTXs from group I to which CTX1B belongs), oxocene (P-CTXs from group II, such as P-CTX3C), and the Caribbean/Indian CTXs [3,4]. CFP was considered a tropical and subtropical water disease; however, recently, it has occurred in new areas such as the Canary Islands (Spain) and Madeira (Portugal) [5,6,7,8,9].

CTXs are the result of chemical transformation of their toxin precursors, which are initially produced by benthic dinoflagellates of the genera *Gambierdiscus* and *Fukuyoa* that settle on macroalgae [10]. These initial toxins are ingested mainly by herbivorous fish when feeding on macroalgae; then, these fish could be predated by carnivorous fish, which subsequently would be predated, and so on, climbing the food chain up to high-order carnivores [11,12]. During this process, the toxin may be biotransformed and accumulated through the food web, reaching risk levels considered hazardous to humans. Some authors have suggested that fish metabolism increases the toxic potential of CTXs, which are changed into other toxic analogues, as supported by direct toxicological evidence on purified P-CTX congeners in mice [13,14,15]. The pathway of this process could implicate p450 enzymes of fish metabolism to eliminate toxins from their tissues by oxidation. However, during this process, more potent congeners of CTXs could result [16] or increase their toxicity after exposure to an acidic environment, such as that of the intestinal tract by acid-catalyzed spiroisomerization [13]. However, this tendency, in which the most toxic CTX is the most oxidized, seems to occur in the P-CTXs from group I (oxopenes) and not in those from group II (oxocenes) [4]. Regardless of the bioaccumulation process through the food web, small fish and herbivorous fish could represent a real risk for human consumption [17,18].

CTXs are rapidly absorbed from the gastrointestinal tract and distributed throughout the body, as animal laboratory research shows [19]. CTXs can link to voltage-gated sodium channels (Na_v_) in fish cells [20], but the physiological mechanisms that allow some fish species to tolerate the toxin effects remain unclear [21]. Moreover, CTX-sensitive Na_v_ have been found in all organs and systems affected by CFP (i.e., brain, skeletal muscle, heart, peripheral nervous system, sensory neurons), as these channels may mediate the symptomatology of CFP [22,23].

Only a few trials have been conducted to understand the transmission, biotransformation, and bioaccumulation of CTXs in fish with large differences in the results. This is probably due to the fish species examined and their ability to tolerate CTX effects [21].

Some initial studies were performed with methodologies that prevented a good comparison, as the CTX used and their concentrations were not well determined. Helfrich and Banner [24], in 1963, fed toxic flesh to the herbivorous fish *Acanthurus xanthopterus*, which was capable of accumulating the toxin, albeit the fish showed no signs of intoxication. Davin et al., in 1986 and 1988 [25,26], fed four species of herbivorous and carnivorous fish different toxic substrates and demonstrated that fish could suffer the toxic effects of CTX, in addition to reporting bioaccumulation. In 1992 [27], Lewis exposed *Gambusia affinis*, a freshwater fish species, to P-CTXs added to water; the fish accumulated CTX and developed strong symptoms preceding death. In 1993, a shoal of *Serranus cabrilla* was fed *Gambierdiscus* cells, and changes in liver function and density were observed [28]. Recent trials have reproduced some of those studies results (regarding symptomatology and toxin burden) and provided additional details about the origin of CTXs, the congeners, and doses used [29,30,31,32]. Some experiments have been performed injecting CTX1B and C-CTX1 in fish larvae to observe toxic effects and fish developmental interference [33,34,35]. According to the literature, no feeding trials have been performed using C-CTXs to emulate its transmission in the food web.

Goldfish is an easy species to maintain and handle in captivity. Goldfish also show a major advantage, as they do not live in the natural marine environment where these toxic microalgae are found. Therefore, all the experimental animals were free of CTXs at the beginning of the experimental design. 

For the reasons given previously, this study aimed to feed adult omnivorous goldfish (*Carassius auratus*), of the *Cypriniudae* family, flesh from an amberjack containing C-CTX1 confirmed by LC–MS/MS, with two objectives: to describe whether high levels of toxin could cause any associated symptomatology in the experimental fish receiving the toxin and to estimate the time required for CTX to accumulate in fish tissues.

## 2. Material and Methods

### 2.1. Experimental Fish Species

Goldfish originally from Asia is a resistant, omnivorous, and brightly colored species, frequently used as ornamental fish kept in domestic tanks [36,37]. This species can resist cold temperatures (close to 2 °C) and hypoxic conditions, also can tolerate brackish water [38].

Goldfish of the common variety were obtained from a commercial fish distributor from Las Palmas de Gran Canaria, Spain. All specimens were >2.5 years of age and had an average body weight of 48.05 ± 11.7 g.

### 2.2. Maintenance of Goldfish

The experimental protocol was approved by the Committee for Animal Welfare of the University of Las Palmas de Gran Canaria and by the Department of Agriculture, Livestock, Fisheries and Water of the Canary Islands Government (code no. OEBA-ULPGC 28/2018).

Fish specimens (n = 28) were randomly separated into two groups (experimental and control) after 30 days of acclimatization in our laboratory in 150-L tanks equipped with two filters and an air stone each. Two replicate tanks were used for each group to correctly set the sampling experiment.

Fish were exposed to a natural photoperiod (11 h light, 13 h darkness) and approximately 23 °C temperature. Water parameters, such as pH, oxygen, ammonia (NH_3_), nitrogen molecules (NO_2_, NO_3_), and dissolved oxygen were measured every three days.

### 2.3. Experimental Model

The omnivorous preference and resistance of *C. auratus* makes this species adequate to be used for analyzing the CTX accumulation in intermediate organisms of the food webs under laboratory conditions; however, the lack of any evolutionary compensatory adaptations should be considered.

The experimental model (Supplementary Data S2) consisted of a dietary exposure of goldfish to *Seriola* sp. raw flesh. *Seriola* sp. was captured in the Canary Islands, naturally contaminated with C-CTX1, as confirmed by LC–MS/MS (0.27 ppb). These data were obtained from the University of Vigo, Spain (methodology described in Supplementary Data S1) [39].

The presence of CTX on this raw flesh was determined by a N2a cell-based assay (CBA), which allowed the quantification of CTX-like toxicity, and may be caused by the action of multiple CTXs congeners [1.1 ng CTX1B equivalents (Eq.) g^−1^ raw flesh, ppb]. Toxin concentration and burden in the muscle of the experimental goldfish group were measured after 1, 8, 15, 29, 36, 43, and 84 days of daily toxic feeding (Table 1). Two fish per group were analyzed (one from each tank) (n = 4), except on sampling days 43 and 84, when only one fish from the experimental group (fish no. 11 and 13, respectively) and two from the control group were sampled.

In addition, one fish (no. 14) was separated from the group on day 43 for a depuration process. The fish was then fed commercial food up to day 102 (S2).

During the experiment, the control goldfish were fed non-toxic *Seriola* sp. raw flesh with CTX concentration < limit of detection/limit of quantification (LOD/LOQ) observed by CBA. These control goldfish were sampled along with experimental fish. The control group was also used as a reference for behavioral assessment and appearance of any symptoms.

### 2.4. Food Preparation

Raw flesh used for experimental dietary exposure was homogenized and supplied to the goldfish in agarose gel (35%) to ensure total consumption. This compound does not add nutrients that may interfere with the interpretation of the results.

Agarose gel (Agarose D-1 Medium EEO, Condalab, Madrid, Spain) was prepared at 3% using deionized water at room temperature and heated in a microwave until it completely melted. The mixture was cooled to approximately 45 °C and mixed with homogenized raw flesh before solidification. The resulting gel was cut into cube-shaped 3–5-mm pieces and stored at −20 °C until use.

The resulting CTX concentration in the prepared food obtained by CBA was 0.714 ppb Eq. of CTX1B.

### 2.5. Toxic Dietary Exposure

In aquaculture, bred goldfish are fed a diet corresponding to 2% of the live weight [40]. In the present study, both groups were daily fed 1.9% of their live weight to accommodate their natural behavior; thus, experimental fish received 0.014 ng CTX1B Eq. g^−1^ of fish weight in their food daily. Each feeding was timed as part of the trial to monitor any variations in their foraging habits. In addition, the fish were observed during the next two hours after exposure to search for possible regurgitations or behavioral changes.

At sixth sampling (day 43), fish no. 11 and 14 showed severe symptoms while feeding; one was then sampled and analyzed (no. 11), and the other (no. 14) was isolated to assess a possible recovery after returning to commercial food and identified as “Detox Fish no. 14”.

During this study, each sampling was performed 24 h post feeding and before any new exposure (Table 1). When a total absence of life signs was confirmed, fish were immediately dissected and flesh was collected for the CTX-like toxicity assay. The extraction was conducted on the sampling day, and extracts were stored at −20 °C until toxin analysis.

### 2.6. Extraction of CTX from Experimental Fish Flesh

The extraction of CTX was performed according to the protocol proposed by Lewis [41], with minor modifications based on our laboratory needs. Briefly, 10 g of fish flesh was cooked at 70 °C for 10 min. Each sample was extracted twice with 20 mL of acetone and homogenized with an Ultra Turrax blender at 17,500× *g*. The supernatant was recovered by centrifugation at 3000× *g* for 10 min at 4 °C. Both supernatants were pooled and filtered through a 0.45 μm PTFE filter and evaporated with a rotary evaporator at 55 °C until dried residue. Liquid/liquid partition was conducted twice with a mix of water and diethyl ether (DEE) (1:4). The DEE fractions were pooled and evaporated to dryness under a N_2_ current. The resulting residue was dissolved for subsequent partitioning in methanol:water (8:2) and N-hexane (1:2). The N-hexane upper phase was discarded, and 4 mL of N-hexane was added to the methanolic fraction for extra cleaning. The methanol phase was collected and dried under N_2_ current at 40 °C. The final residue was re-dissolved in 4 mL of methanol and preserved at −20 °C until analysis with CBA.

### 2.7. Determination

A mammalian CBA was the selected method for this study because it enables the estimation of the activity caused by multiple CTX congeners, which may be present in the sample from the *Seriola* sp. raw fish diet or resulting from the goldfish metabolism. This method of analysis also allows the quantification of this toxic activity [42].

The cellular line used in this study was the Neuro-2a (Cell line: CCL131, from ATCC, LGC Standards SLU, Barcelona, Spain) and cells were maintained in Roswell Park Memorial Institute medium (RPMI)-1640 supplemented with 5–10% of fetal bovine serum at 37 °C in a 5% CO_2_ atmosphere. The Pacific type 1 CTX standard (STD) (named P-CTX-1) was provided by Pr. Richard J. Lewis (Queensland University, Australia) [43] and used for the assessment of CTX-like toxicity by CBA.

The cytotoxicity assay was conducted as previously described by Caillaud et al. [44] with minor adaptations; cells were seeded in a 96-well flat-bottom plate (200 µL/well) at a concentration of 40,000 cells/well. Ouabain (0.1 mM) and veratridine (0.01 mM) were used to assess cell mortality in the presence of CTX. After incubation, cells were exposed to goldfish flesh extract and the CTX1B STD at decreasing concentrations. Each sample extract and the STD were assayed in triplicate wells, and each value was taken from the average of these three absorbances.

Cell viability was evaluated using MTT [3-(4,5-dimethylthiazol-2-yL)-2,5-diphenyltetrazolium] and DMSO solutions. Absorbances were read at 570 nm with a multi-well spectrophotometer scanner; dose–response curves were evaluated with Microsoft Office Excel 2016 and GraphPad Prism 7 software (GraphPad, San Diego, CA, USA).

CTX-toxicity levels in goldfish flesh were determined twice by comparison with the standard curve of CTX1B (IC_50_ = 3.257 ± 0.149 pg CTX1B mL^−1^), obtained the same day as the corresponding assay. A response producing less than 20% cell mortality was considered a non-toxic effect [44]. The LOD and LOQ were set at the concentration of CTX1B STD, causing 20% inhibition of cell viability (IC_20_) considering the maximum concentration of fish extracts for cell exposure. According to the mean value of IC_20_ (1.528 ± 0.045 pg CTX1B mL^−1^) observed in the dose–response curve obtained with the STD and the maximum concentration of extracts (150 mg tissue Eq. mL^−1^), the LOD/LOQ obtained was 0.010 ng CTX1B Eq. g^−1^ of goldfish flesh (ppb).

CTX-1B was used as the reference standard to evaluate toxicity, as our laboratory regularly analyses all fish by using this standard. As in all toxicological studies, it is important to use a reliable standard to compare toxicity in different samples, regardless of the toxins they may contain.

## 3. Results

### 3.1. Symptomatology and Fish Behavior

Experimental goldfish (n = 14) were exposed to a constant daily level of CTX during this experiment (84 days). Within the first two weeks, experimental fish displayed no symptoms or abnormal behavior (Table 1). However, after that period, all the exposed specimens progressively developed symptomatology and some behavioral disturbances, such as loss of brightness and lethargy (which disappeared during feeding). Two goldfish, no. 11 and 14, showed signs of intoxication two weeks after lethargy onset, with rapid evolution and deterioration. Thus, the symptoms that appeared during feeding time included some loss of equilibrium (sideway swimming) and reduced stability (drifting in the water column). After two days, symptomatology evolved to hyperactive signs, such as intermittent erratic swimming with jerking movements, disorientation, and difficulty on feeding. On day 43, goldfish no. 11 was sampled and analyzed by CBA, and Detox Fish no. 14 was maintained alive to evaluate a possible recovery of signs after returning to commercial food.

On day 58, one of the two last fish (no. 12) started to show signs similar to those described for the two goldfish mentioned previously. In 24 h, fish no. 12 developed an inability to feed, which was concomitant to continuous excitation and loss of control to avoid fixed objects, such as filters, air stones, or thermometers. After two days without feeding and no changes in symptomatology, the fish was sampled. The necropsy revealed a compressive tumor lesion in the cerebellum, which, along its variable and deficient feeding behavior, makes the estimation of the CTX toxicity difficult to assess; thus, this result was not included in the present survey.

The last experimental fish (no. 13) failed to show strong symptoms; however, it showed the same symptoms as previous specimens (some lethargy and loss of brightness). This fish was fed a toxic diet until day 84, at which time it started to refuse food. To evaluate an appropriate eating capacity, the fish was fed commercial granulated food (2 mm larger than toxic food), which was correctly ingested; but the toxic diet offered the next day was refused again. The CTX-intake experimental trial was then considered to be completed, and the level of CTX in that specimen was evaluated.

Detox Fish no. 14, which did not receive toxic food after day 43 to evaluate CTX recovery, was fed commercial food for 60 more days. The strong symptomatology appeared during feeding time in the first two days after returning to commercial food. This condition started to fade, but lethargy remained. Nevertheless, it was not until day seven of regular feeding that lethargy began to disappear and, on day 20, that color and behavior was completely recovered to normal.

Control goldfish fed non-toxic food did not show atypical behavior. Additionally, several fish sampled from this group showed gonadal development, and one female was near spawning. Although experimental goldfish fed a toxic diet were exposed to the same environmental conditions, they never showed any reproductive behavior.

### 3.2. CTX Accumulation Profile in Goldfish Muscle

**Figure 1:**
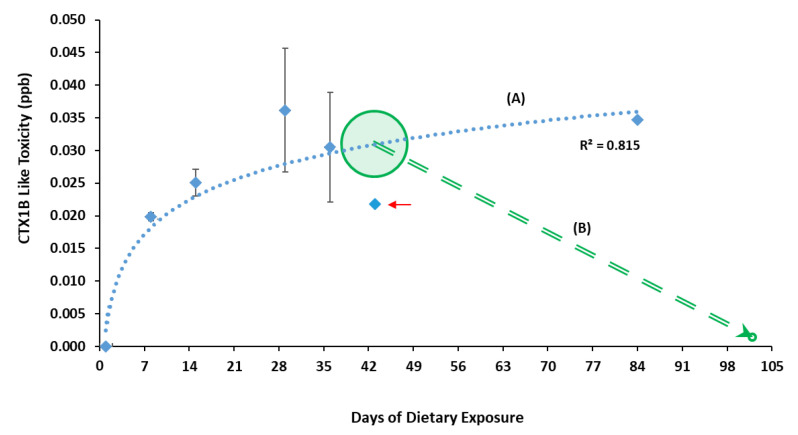
The mean ciguatoxins CTX-like toxicity level (expressed in CTX1B ppb) in fish muscle per day of sampling (1, 8, 15, 29, 36, 43, and 84) measured by cell-based assay CBA. (**A**) Logarithm regression with coefficient R^2^ = 0.815; arrow shows fish no. 11, which displayed hyperactive symptomatology with difficulty in feeding, days before sampling. (**B**) Expectable CTX concentration for Detox Fish no. 14 from the moment of suspending toxic feeding on day 43 (highlighted circle), and the CBA result obtained on day 102, after returning to commercial food feeding.

CTX presence in muscle tissue from the goldfish was evidenced by the CBA and estimated by comparing it with a CTX1B STD [45]. The CTX-like toxicity in the flesh was established with the assay 24 h after the first feeding with a LOD/LOQ of 0.010 ng CTX1B Eq. g^−1^ of goldfish flesh and reached quantifiable levels over this limit from the second sampling, performed on day eight, with a greater value than the feeding rate (0.020 ± 0.001 ng CTX1B Eq. g^−1^ of fish flesh; Figure 1). Levels of CTX in muscle reached the risk value specified by the European Food Safety Authority (EFSA) (0.01 ng CTX1B Eq. g^−1^; Table 2) [46], in eight days of daily intake with toxic food. The CTX levels in fish muscle increased at a slow rate during the following weeks until day 29, when the concentration seemed to stabilize until day 84 (~0.03 ng CTX1B Eq. g^−1^), showing a positive correlation between days of dietary exposure and CTX-like toxicity, with a coefficient of determination (R^2^) of 0.815 for a logarithmic regression (Figure 1). This includes fish no. 11, which presented difficulties in feeding due to its symptoms, and was sampled on day 43, resulting in 0.022 ng CTX1B Eq. g^−1^ (Figure 1).

Fish no. 14 allowed the study of possible CTX detoxification. The fish was sampled 60 days after suspending the toxic feed on day 43 and did not show any CTX-like toxicity (Figure 1).

**Figure 2:**
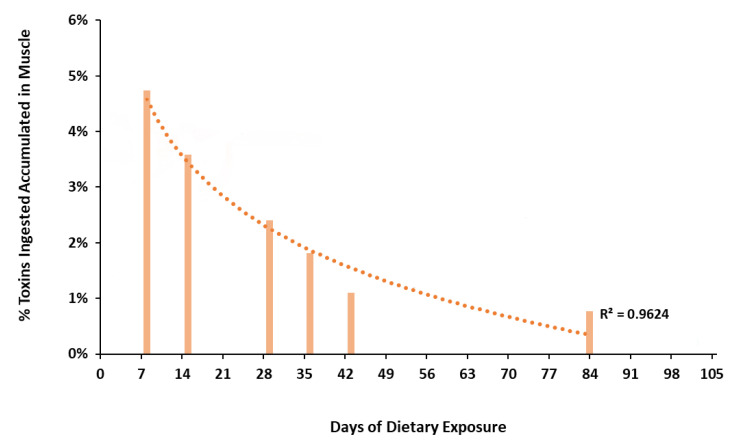
Percentage of CTX muscle burden (%) compared to the total toxin ingested over the experiment per day of sampling (logarithmic regression with a coefficient R^2^ = 0.9624).

The percentage of ingested toxins accumulated in muscle was calculated for each individual and adjusted by the days of daily intake and the toxin concentrations in the muscle. This percentage was not constant, showing a progressive decrease from 4.7% of the total toxin intake accumulation (day 8) to 0.7% of the total toxin ingested at the end of the experiment (day 84). These data displayed a positive correlation between days of exposure and percentage of CTX accumulated in muscle with an R^2^ of 0.9624 (Figure 2 and Table 2).

## 4. Discussion

### 4.1. Behavioral Disturbances and Signs of Intoxication

Experimental goldfish were asymptomatic to CTX until day 15 of daily toxic feeding (0.014 ng CTX1B Eq. g^−1^ of fish flesh), when they started to show lethargy and color brightness alteration. These hypoactive behaviors after continuous toxic intake in fish have also been reported in previous studies [25,26,27,29]. Ledreux et al. [29] fed herbivorous *Mugil cephalus* (improbably exposed to CTX in its natural habitat) *Gambierdiscus polyniensis* cells (0.3 ng CTX3C Eq. g^−1^ of fish); in that study, behavioral disturbance was more evident on the second consecutive day of feeding (6 ng CTX3C fish^−1^ of cumulative dose intake). Clausing et al. [30] fed an *Acanthuridae* fish (*Naso brevirostris,* an herbivorous fish naturally present in CFP endemic areas), similar toxin daily doses (0.4 ng CTX3C Eq. g^−1^ of fish), but the fish did not show any symptoms or behavioral disturbances. As evidenced by an earlier survey, in 1958, another *Acanthuridae* fish fed toxic flesh from *Lutjanus bohar* did not show any symptoms [24]. The present study suggests that goldfish may be particularly sensitive to dietary CTX.

Lethargy and alteration in skin color were the first and main intoxication effects displayed by all the experimental goldfish until the end of the present trial. Davin et al. [25] observed these two symptoms at the beginning of their experiment with *Thalassoma bifasciatum* fed *Gambierdiscus* sp. cells collected in the Caribbean Sea, which were also observed by Lewis in 1992 [27]. Additionally, in the present study, two goldfish (no. 11 and 14) displayed hyperactive symptomatology, such as erratic swimming episodes and jerking. Goldfish no. 11 was sampled on day 43, and a lower CTX concentration was displayed in muscle (0.022 ppb CTX1B Eq.) than those measured previously, day 36 (0.031 ppb CTX1B Eq.) and subsequent day 84 (0.035 ppb CTX1B Eq.; Table 2, Figure 1). This unexpected value may be associated with difficulty on food intake during the days before sampling and ability of detoxification, as it was proved with Detox Fish no. 14 after 43 days of cumulative toxic feeds and subsequent 60 days of regular feeding. However, this suggestion does not overlook individual susceptibility and a possible chronic effect on the nervous system in these fish, unrelated to the actual amount of CTXs in muscle tissue, as this toxin could bind to the fish brain Na_v_ [20]. Detox Fish no. 14 showed hyperactive symptoms during the two days after returning to commercial feeding and, seven days later, these symptoms started to disappear. This fish completely recovered at day 20. Similar findings were obtained by Davin et al. [26], studying *Lutjanus apodus* fed barracuda ether extract after a short period of toxic intake. The symptoms displayed (disequilibrium, erratic behavior, hitting the sides and bottom of the tank while swimming) disappeared after 94 h of the last exposure, proving the capacity of fish to recover from ciguatoxic effects. Detox Fish no. 14 maintained normal behaviur and healthy appearance as the control goldfish until sampling (60 days after toxic food was suppressed). This fact seems to indicate that goldfish takes less than 60 days to remove CTXs from the muscle tissue, reaching non-detectable levels.

Experimental goldfish, in addition to showing signs of intoxication, also showed differences in breeding behavior and gonadal development compared with control fish. Several experiments performed in *Oryzias* spp. larvae or embryos exposed to P-CTX and C-CTX demonstrated that CTX effects interfere with species survival due to abnormalities and malfunction in fish larvae [33,34,35,47]. In the case of the reproductive effects of CTX in adult fish, a recent study performed with *Oryzias melastigma* showed that the ingestion of CTX1B can cause, apart from various symptoms, a decline in egg production, hatching failure, and delay of hatching, which results in a decrease in reproductive success [31]. This would explain the difference in gonadal development between the experimental and control groups. However, further investigation is needed.

The fact that the last experimental goldfish (no. 13; day 84) gradually increased feeding time (offering the same amount of food) and consciously rejecting the toxic food might suggest that the fish could recognize the harmfulness of this diet. Thus, this behavior could be a survival strategy in some fish species naturally exposed to CTXs as a conditioned response, similar to observations described for other animal species with foodborne diseases [48] and, particularly in goldfish, with a similar progression [49]. If this occurs in nature, as other authors suggest [26], it will allow the total recovery of the fish species, thereby allowing them to hide from their predators for survival. However, this hypothesis needs further studies to be refuted or confirmed.

### 4.2. CTX Accumulation in Muscle Tissue

Some aspects of C-CTXs are not yet well known, such as their producer, *Gambierdiscus* sp. [4]. However, attending to some of the chemical similarities with P-CTXs of group II, C-CTX could accumulate in the goldfish tissue and be transferred to a possible predator with no major chemical modifications [29]. Interestingly, goldfish are stomatchless fish with a stable pH (6.6–8.4) throughout its gut when exposed to a carnivorous diet [50]. Acid digestive tracts could promote the production of polar forms of CTX, increasing their toxicity by acid-catalyzed spiroisomerization [13]. A recently published report [3] suggests that reliable toxicity equivalency factors for CTX-group toxins could not be derived because of the limited data from in vivo assays in mice (MBA) [43,46,51]. However, another recent report [52] provides a comparison of the toxicity by CBA of C-CTX1 and CTX3C in relation to CTX1B, which is two times more potent than the other two. This approach theoretically allows comparison of our results with the few trials performed with fish fed CTX3C congeners (P-CTX group II) [29,30].

Thus, experimental goldfish ingested an average of 0.68 ng CTX1B Eq. per individual (0.014 ng CTX1B, Eq. g^−1^ of fish) in the first intake, and neither was CTX-like toxicity evidenced by CBA (LOD/LOQ = 0.0104 ppb), nor were signs of intoxication observed. However, Ledreux et al. [29] fed their experimental fish 0.3 ng CTX3C Eq. g^−1^ of fish, and the authors observed CTX activity at 24 h post exposure. In addition, their experimental fish never showed an increase in CTX retention after consecutive doses; nevertheless, the fish showed signs that were more evident on the second day of feeding, similar to those presented by goldfish since day 15. These differences could be associated with the metabolism of each fish species to clean exogenous molecules from their tissues, the different susceptibility, and food sources. Additionally, goldfish could store the toxins in other organs, such as liver or gonads, at a higher rate than in muscle, as observed in other fish species [29,32,53,54], although this fact was not demonstrated in this work.

Since the second measurement on day 8, goldfish muscle started to show CTX-like toxicity (0.020 ng CTX1B Eq. g^−1^ of muscle), which represents 4.7% of the total toxin ingested, but no signs of intoxication were observed. The symptomatology occurred from day 15, with an initial concentration of 0.025 ng CTX1B Eq. g^−1^ of muscle (after the intake of 1.17 ng CTX1B Eq. per fish and 0.41 ng CTX1B accumulated in the muscle tissue). This CTX ingestion could represent a threshold below which the goldfish remain asymptomatic. The symptomatology presented by the goldfish beyond that level of CTX may favor predation, as Davin et al [25] suggested. This initial resistance may allow wild fish to accumulate high amounts of CTX in their muscle before reaching large carnivores. This finding is in accordance with the *N. brevirostris* experiment, in which CTXs reached large amounts in the fish tissues, without clinical signs [30].

In 2018, Clausing et al. [30] found a linear increase in CTX burden in muscles from *N. brevirostris* juveniles during their experiment. However, the toxin concentration in muscle increased during the first eight weeks and then remained stable, according to the different growth rates of muscle assessed during their experiment. The goldfish used in this study were adults between two and three years old with a small growth rate and no great variations in muscle volume during the experiment. Thus, the different toxin evaluations observed in goldfish could be explained by an increase in the toxin depuration rate by continuous exposure or the impossibility of fixing more CTX in muscle tissue due to the saturation limit (Figure 1 and Figure 2).

Regarding Detox Fish no 14, due to the destructive character of the sampling, the exact CTX amount in the muscle tissue was not possible to be determined at the time of returning to commercial food (day 43); however, it was expected to be 0.022–0.03 ng CTX1B Eq. mL^−1^ (Figure 1, line B). Additionally, the fish did not show any CTX-like toxicity 60 days after suspending the toxic diet when sampling was performed. This result provides evidence of a depuration process in the goldfish muscle tissue.

Depuration of the assimilated CTX in goldfish muscle and recovery from symptomatology is evident from our results, as other authors [26,32] observed in other fish species. However, further studies should be performed to clarify the rapid metabolic process involved in detoxification and the mechanisms to avoid toxic effects in fish species that can store high concentrations of CTXs in their tissues for long periods [11,27].

## 5. Conclusions

For the first time, goldfish (*C. auratus*) has been described as a fish species with the capability to accumulate C-CTX1 in its muscle tissue and suffer its toxic effects.

These results suggest that goldfish could recover from the toxic effects caused by CTXs and eliminate them from muscle tissue.

The data obtained in this study propose goldfish as an efficient model to better study CTX accumulation and depuration processes in fish, even though goldfish is not a marine species.

## Figures and Tables

**Table 1:** Individualized fish data from the experimental group, collected on each sampling day.

Experimental Fish (No)	Sampling Day ^a^	Consecutive Dose Intakes ^b^	Body Weight ^c^	Muscle Weight ^d^	Standard Length ^e^	Observations
1	1	1	53.7	13.1	11.7	Normal behavior
2	1	1	46.9	11.4	11.5	Normal behavior
3	8	8	46.0	11.3	11.0	Normal behavior
4	8	8	43.7	11.6	11.0	Normal behavior
5	15	15	55.5	15.1	11.8	Lethargy and loss of brightness
6	15	15	59.4	17.8	12.5	Lethargy and loss of brightness
7	29	29	42.8	11.2	11.0	Lethargy and loss of brightness
8	29	29	63.3	15.8	12.5	Lethargy and loss of brightness
9	36	36	81.2	21.4	13.5	Lethargy and loss of brightness
10	36	36	41.2	12.0	10.9	Lethargy and loss of brightness
11	43	43	38.3	10.6	10.2	Sideway swimming, drifting, jerking movements and difficulty on feeding
12	60	57	38.3	10.0	10.0	Starvation for 3 days and discarded after observing a tumor lesion in the cerebellum
13	84	82	45.4	10.6	11.0	It began to increase feeding time and then consciously refused toxic feeding on the last 2 days
14	102	43	45.6	10.5	10.9	Same symptomatology than fish no. 11 on day 43, then, it was separated and sampled after 60 days of commercial food feeding

^a^ Sampling performed >24 h after last feeding. ^b^ Total daily doses ingested by each goldfish before sampling. ^c^ Total body weight (g). ^d^ Total weight of muscle tissue collected (g). ^e^ Measure taken from mouth up-the peduncle (cm).

**Table 2:** Comparison of the Ciguatoxin (CTX) values ingested by goldfish and detected in muscle tissue during sampling.

Sampling Day	Experimental Fish Sampled (No.)	Mean Total Quantity of Toxin Ingested ^a^ (pg CTX)	Mean Muscle CTX-Like Toxicity Level ^b^ (ppb CTX)	Mean Muscle Toxin Burden ^c^ (pg CTX)	% Toxins Ingested Accumulated in Muscle ^d^
1	1, 2	681.82	0.000 ± 0.000	0.00 ± 0.00	0.0%
8	3, 4	4864.55	0.020 ± 0.001	226.92 ± 10.90	4.7%
15	5, 6	1168.13	0.025 ± 0.002	411.09 ± 13.81	3.5%
29	7, 8	20,848.21	0.036 ± 0.010	504.36 ± 246.93	2.3%
36	9, 10	29,877.93	0.031 ± 0.008	482.46 ± 63.09	1.8%
43	11	22,334.22 *	0.022	231.59	1.0%
84	13	51,726.92 *	0.035	368.17	0.7%

^a^ Calculated as the total sum of CTX ingested by fish until sampling time, divided by the number of fish sampled on that day. ^b^ The average CTX-like toxicity level per sampling day determined by CBA was conducted twice in each fish sample. ^c^ Calculated by multiplying the CTX concentration by the weight of muscle tissue collected per fish. ^d^ Calculated using total quantity of toxin ingested and muscle toxin burden per sampling day. * Only one data point is available within the sampling day.

## Data Availability

The data presented in this study are available on request from the corresponding author.

## Supplementary Materials

The following are available online at https://www.mdpi.com/2076-2615/11/1/242/s1. Data—Chromatogram obtained after the LC-MS/MS analysis in MRM mode and for Supplementary data S2—Outlined research design.
