# Supplementary material for: Accumulation of C-CTX1 in Muscle Tissue of Goldfish (Carassius auratus) by Dietary Experience
Source: Animals (Basel). 2021 Jan 19;11(1):242. doi: 10.3390/ani11010242 (PMC7835822; doi:10.3390/ani11010242)

Supplementary data S1

Chromatogram obtained after the LC-MS/MS analysis [1] in MRM mode of **A)** C-CTX1 pure standard (5 ng / mL); **B)** C-CTX1 detected in the *Seriola* sp. Along with the C-CTX1 peak, some traces of C-CTX1-Me and interferences, which could be erroneously identified as other CTXs from the P-CTXs family, were found.

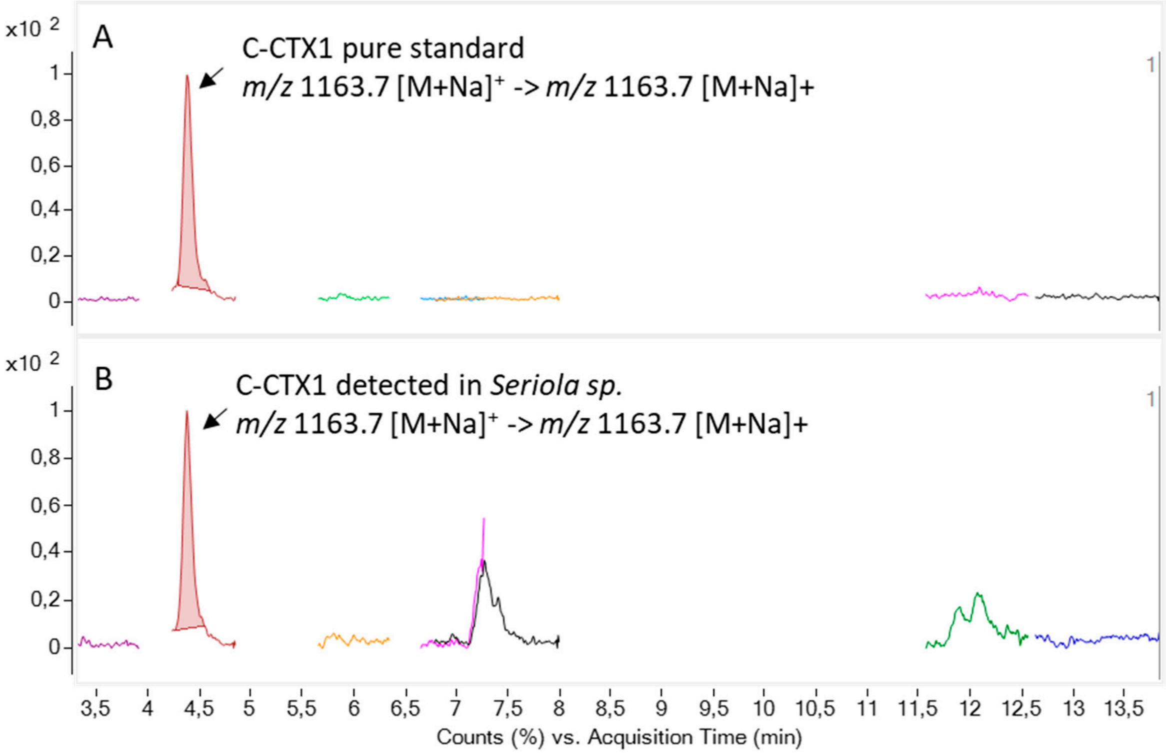

Supplementary data S2

Outlined research design.

**Objectives**	To describe the symptomatology in goldfish (*Carassius auratus*) associated to ciguatoxin (CTX) ingestion.
	To estimate the time required for CTXs to be accumulated in fish muscle.
**Fish species**	Goldfish which lives in a naturally CTX free environment.

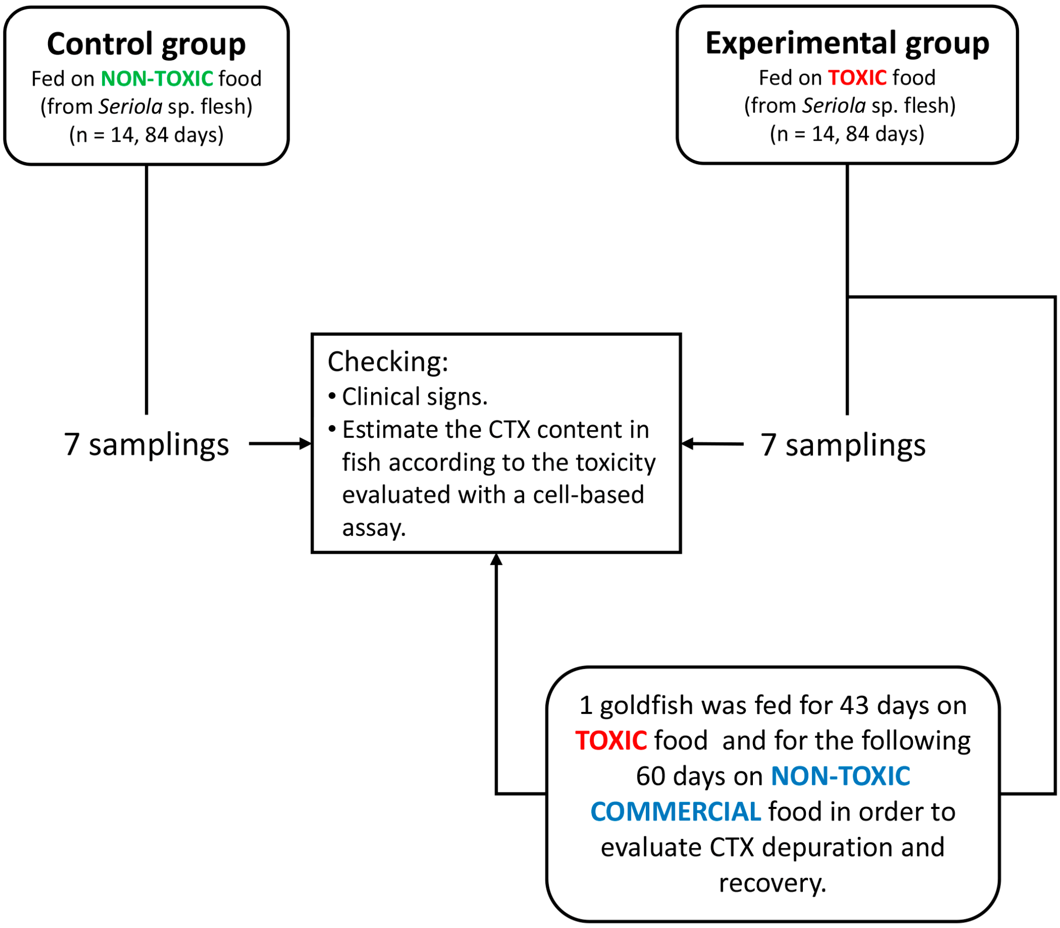

Supplement: Supplementary file 1 [file animals-11-00242-s001.pdf]
